# Cornea lenticule viability and structural integrity after refractive lenticule extraction (ReLEx) and cryopreservation

**Published:** 2011-12-28

**Authors:** Karim Mohamed-Noriega, Kah-Peng Toh, Rebekah Poh, Deepashree Balehosur, Andri Riau, Hla M. Htoon, Gary S.L. Peh, Shyam S. Chaurasia, Donald T. Tan, Jodhbir S. Mehta

**Affiliations:** 1 Tissue Engineering and Stem Cell Group, Singapore Eye Research Institute, Singapore; 2 Singapore National Eye Centre, Singapore; 3 Singapore Eye Research Institute, Singapore; 4 Office of Clinical Sciences, Centre for Quantitative Medicine: Duke-NUS Graduate Medical School, Singapore; 5 Department of Ophthalmology, Yong Loo Lin School of Medicine, National University of Singapore, Singapore; 6 Department of Clinical Sciences, Duke-NUS Graduate Medical School, Singapore

## Abstract

**Purpose:**

To assess and compare keratocyte viability and collagen structure in cornea stroma lenticules collected immediately after refractive lenticule extraction (ReLEx) and one month after cryopreservation.

**Methods:**

The fresh and cryopreserved human stroma lenticules procured after ReLEx were processed for ultrastructural analysis of keratocytes and collagen fibrils with transmission electron microscopy (TEM), apoptotic cell detection with deoxynucleotidyl transferase-mediated nick end labeling assay (TUNEL) assay, and cultured for keratocyte-specific gene expression analysis using reverse transcriptase polymerase chain reaction (RT–PCR).

**Results:**

The periphery of the lenticule had greater TUNEL-positive cells compared to the center of the lenticule in both fresh and cryopreserved groups. There was an increase in TUNEL-positive cells after cryopreservation, which was significantly higher in the center of the lenticule, but not in the periphery. TEM showed apoptotic, necrotic and viable quiescent keratocytes in fresh and cryopreserved lenticules. Collagen analysis with TEM showed a well preserved and well aligned structure in fresh and cryopreserved lenticules; without significant change in the total number of collagen fibrils but with an increased collagen fibril density (CFD) after cryopreservation. In vitro, isolated keratocytes derived from fresh and cryopreserved lenticules exhibited a typical fibroblastic phenotype. RT–PCR showed a positive gene expression for keratocan (*KERA*) and aldehyde dehydrogenase 3A1 (*ALDH3A1*) in cells isolated from fresh and cryopreserved lenticules.

**Conclusions:**

The stromal lenticules extracted from ReLEx surgery remain viable after cryopreservation. Although they showed a decrease in CFD, the collagen architecture was preserved and there was good cellular viability.

## Introduction

Laser in situ keratomileusis (LASIK) is a refractive surgery procedure involving the creation of a corneal flap and corneal reshaping by an excimer laser. Femtosecond (FS) lasers have revolutionized this procedure by enabling the non-mechanical creation of corneal flaps [1,2]. Femtosecond-LASIK (FS-LASIK) has been shown to have many advantages over mechanical microkeratome-LASIK (MK-LASIK). They create more predictable flaps with respect to flap thickness, diameter and hinge width [3-7]. FS laser flaps have greater biomechanical stability and stronger flap adherence [5,6]. Furthermore, FS lasers create a smoother stromal bed surface with less surgically induced astigmatism, less higher order aberrations, and better postoperative contrast sensitivity compared to MK-LASIK [3-6]. Recently, we have shown that there is less intraocular pressure fluctuations during FS-LASIK compared to MK-LASIK [6].

The FS laser is an infrared, neodymium-doped yttrium aluminum garnet (Nd:YAG) laser. Ultra-short pulses of light at high repetition rates elicit a cleavage of tissue planes by creating small cavitation bubbles, thus making highly accurate incisions with minimal tissue damage [1,2]. A new refractive lenticule extraction (ReLEx) procedure is the first all-in-one FS-laser refractive procedure aimed for the correction of myopia/myopic astigmatism by creating a refractive intrastromal corneal lenticule [8-10]. Currently, ReLEx can only be performed with the VisuMax® FS laser system (Carl Zeiss Meditec, Jena, Germany). ReLEx encompasses two different techniques; (i) femtosecond lenticule extraction (FLEx), that requires the creation of a flap akin to LASIK with an additional posterior cut to create a stromal lenticule, which is then removed manually and (ii) small incision lenticule extraction (SMILE) where there is no flap, instead the lenticule is extracted through a small arcuate incision [8-11].

ReLEx is a new refractive procedure yet to be approved by the FDA, but with recent reports show promising results that are comparable to FS-LASIK. A prospective 12 month follow-up study of 62 eyes with myopic astigmatism shows a mean residual refractive error of 0.15±0.46 D and a mean UCVA of 1.10±0.26 [9]. A prospective 6 months follow up study of 108 eyes with myopia showed a mean SEM (spherical equivalent) of −0.19±0.47 D and UCVA of 20/40 or better in 97% of cases [10].

ReLEx may have certain advantages over FS-LASIK as only one laser is used [8]. Recently, we have shown that there is less inflammation at higher corrections of myopia with ReLEx compared to FS-LASIK [12]. This may relate to the fact that ReLEx utilizes the same energy to create a lenticule irrespective of refractive error, whereas in LASIK treatment time and energy increases with higher refractive corrections [12]. A significant potential advantage of the SMILE form of ReLEx surgery lies in the flapless nature of the procedure whereby the lenticule is extracted through a small pocket incision, obviating most flap-related complications and possibly causing less postoperative discomfort, neurotrophic status and dry eye.

Another advantage of ReLEx is that it could be a reversible refractive procedure. The removal of the refractive intrastromal lenticule in situ allows the possibility of reimplantation at a future date. To achieve this, the integrity of the extracted stroma lenticule must be maintained after long-term storage. Previous studies have shown that corneal tissue can be stored using cryopreservation [13-15]. However, the process of freezing and thawing can damage cornea endothelium [15-19] and stroma, of which only the latter is relevant in ReLEx [15,19].

The aim of the present study was to assess the viability and collagen architecture of the extracted human cornea lenticule following ReLEx procedure and storage of the stromal lenticule using a cryopreservation technique.

## Methods

### Human cornea samples

Twelve human cadaveric corneas stored in Optisol medium were obtained from the Lions Eye bank (Tampa, FL). The mean donor age was 60±10 years (range: 48–74); the death-to-tissue harvest time was 24 h or less in all cases and the mean time from death to surgery was 10±5 days (range: 5–17). All studies related to human tissues were approved by the Institutional Review Board of Singapore Eye Research Institute and Singapore National Eye Center.

### Experimental design

**Figure 1:**
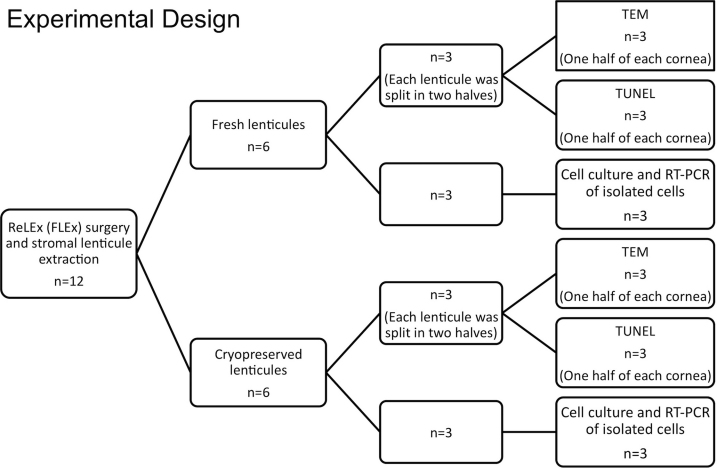
Flow diagram showing the experimental design. ReLEx: Refractive lenticule extraction. FLEx: Femtosecond lenticule extraction. RT–PCR: reverse transcription-polymerase chain reaction. TEM: Transmission electron microscopy. TUNEL: deoxynucleotidyl transferase-mediated nick end labeling assay.

The flow diagram for the detailed experimental plan is described in Figure 1. Briefly, ReLEx FLEx surgery was performed on 12 corneas with a −9.0 diopters spherical treatment and the extracted stromal lenticules were divided into two groups of 6 lenticules each. One group was analyzed immediately after extraction (fresh group) and the other group was cryopreserved for a period of one month (cryopreserved group). Three lenticules from each group were used for cell culture to analyze gene expression of isolated cells using reverse transcription-polymerase chain reaction (RT–PCR). The three remaining lenticules from each group were subsequently cut into half. One half was used for tansmission electron microscopy (TEM) and other was used for deoxynucleotidyl transferase-mediated nick end labeling (TUNEL) assay.

### Refractive lenticule extraction (ReLEx) procedure

**Figure 2:**
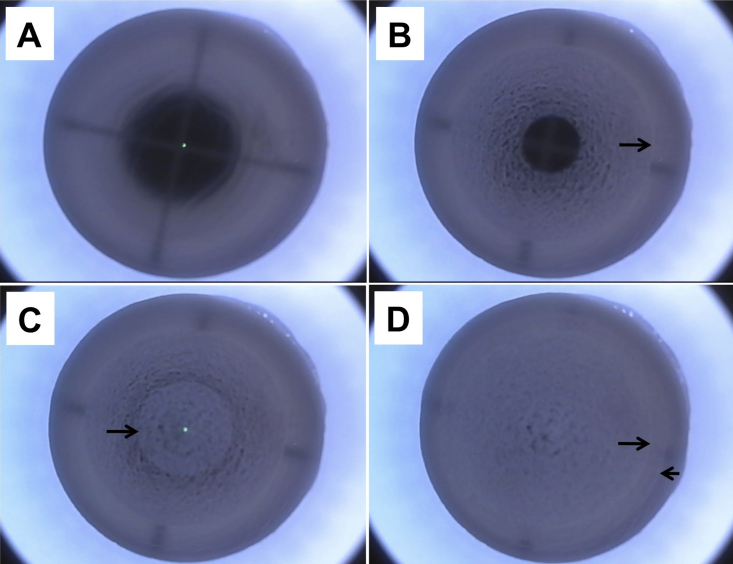
Laser scanning pattern and incisions created during femtosecond lenticule extraction (FLEx) procedure in the human cornea. **A**-**D**: Representative images of specific steps. **A**: Before the delivery of the laser the eye must be centered and full suction must be achieved. **B**: Creation of the posterior surface of the refractive lenticule with a scanning pattern in a centripetal direction (spiral in, arrow). **C**: Creation of the anterior surface of the refractive lenticule with a scanning pattern in a centrifugal direction (spiral out, arrow). **D**: Edge of the lenticule (long arrow) and creation of the periphery of the flap (short arrow).

**Figure 3:**
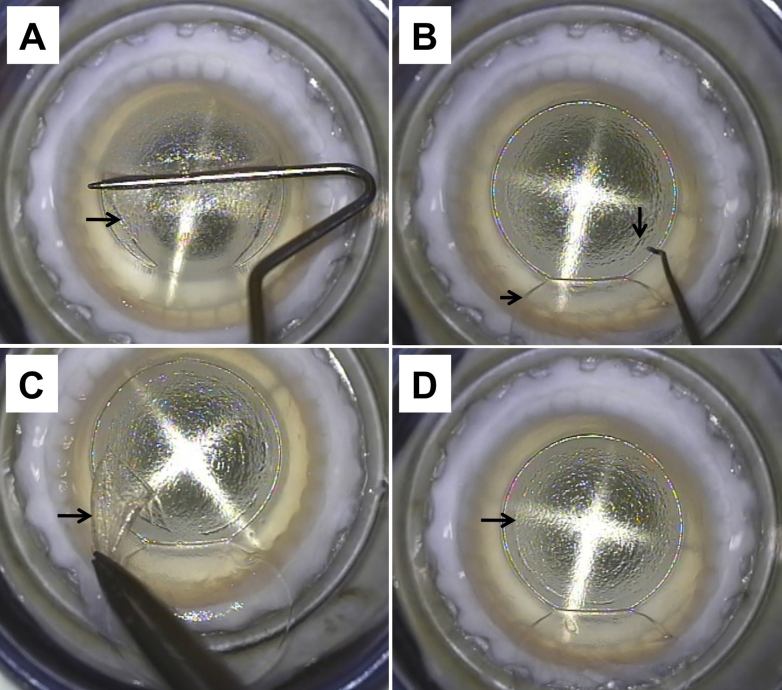
Surgical steps in Femtosecond lenticule extraction (FLEx) **A**: Dissection, opening and lifting of the flap (arrow). **B**: Once the flap (short arrow) is flipped, the edge of the lenticule (long arrow) and the plane of the posterior surface of the lenticule are identified. **C**: The lenticule (arrow) is separated and removed from the cornea. **D**: After extraction of the lenticule, the previous location of the lenticule edge (arrow) can be identified in the surface of the stromal bed just before the flap is repositioned.

ReLEx procedure (FLEx technique) was performed by the same surgeon (J.S.M.) using a 500 kHz Femtosecond (FS) laser (VisuMax; Carl Zeiss Meditec, Jena, Germany; Figure 2 and Figure 3) as described earlier [11,12]. Briefly, the corneoscleral buttons were mounted on an artificial anterior chamber (AAC; Coronet Network Medical Products, Yorkshire, UK) and attached to an infusion bottle via a 3-way tap to maintain physiologic pressure. We first centered the AAC using the microscope internal light so that the fixation lights was in the middle of the cross-hair of the AAC. A small (S) VisuMax curved interface cone was used and the procedure was performed once full suction was achieved. The refractive and non-refractive femtosecond incisions were as follow. First, the posterior surface of the refractive lenticule (spiral in), and the lenticule side cuts were created. Second, the anterior surface of the refractive lenticule (spiral out) was formed which was then extended centrifugally to form the periphery of the flap and followed by the flap side cut (Figure 2). The laser parameters were; flap thickness: 120 µm, flap diameter: 7.5 mm, lenticule diameter: 6.5 mm, lenticule side cut 15 µm and cut angle 90° and a refractive correction of −9 diopters. Power of 130 nj, spot distance and tracking spacing: 3 µm for lenticule and flap, 2.5 µm for lenticule side cut and 2 µm for flap side cut. After suction was released, a Siebel spatula (Rhein Medical, Heidelberg, Germany) was inserted under the flap near the hinge; the flap was opened and lifted. The edge of the refractive lenticule was separated from the stromal bed with a sinsky hook and the posterior border of the lenticule gently separated with the Siebel spatula. The lenticule was grasped and removed with traumatic serrated forceps and the flap was repositioned (Figure 3). The extracted lenticule was either processed immediately or processed for cryopreservation.

### Cryopreservation and thawing of stromal lenticule

Extracted lenticules were washed twice (10 min each) in a PBS buffered antibiotic/antimycotic solution. The lenticules were then transferred into a cryovial and resuspended in 500 μl medium containing 10% FBS. A stock freezing solution containing 10% FBS and 20% dimethyl sulfoxide (DMSO; Sigma, St. Louis, MO.) was added slowly in a drop wise manner to an equal volume ratio, to make a final volume of 1 ml freezing solution containing 10% FBS and 10% DMSO. Freezing of the cryovial containing the stromal lenticule was performed at a controlled cooling rate with a cryo-container (“Mr. Frosty”; Thermo Fisher Scientific, Roskilde, Denmark) in a −80 °C freezer overnight, and transferred into liquid nitrogen the following day for long-term storage (one month).

After one month of cryopreservation, the vial containing the frozen stromal lenticule was rapidly thawed in a water bath at 37 °C and rinsed twice in a PBS solution to remove cryoprotectant agents. Thawed lenticules were either enzymatically digested for cell culture and gene expression or fixed for TEM analysis and TUNEL assay.

### Ultrastructural analysis

Transmission electron microscopy (TEM) was performed in fresh (n=3) and cryopreserved (n=3) groups for the analysis of keratocyte viability and collagen architecture. Lenticules were fixed with 2% paraformaldehyde and 2% glutaraldehyde in 0.1 M sodium cacodylate buffer, pH 7.4 (Electron Microscopy Sciences, Hatfield, PA) at 4 °C overnight. They were washed in sodium cacodylate buffer, rinsed with distilled water and trimmed into smaller pieces. Tissues were then post-fixed in 1% osmium tetroxide and potassium ferrocyanide (Electron Microscopy Sciences) to enhance membrane contrast. After extensive rinsing with distilled water, tissues were dehydrated in a graded series of ethanol, and embedded in Araldite (Electron Microscopy Sciences). The ultra-thin sections of 70–90 nm thickness were cut with a Reichert-Jung Ultracut E Ultramicrotome (C. Reichert Optische Werke AG, Vienna, Austria), and were collected on copper grids, double stained with uranyl acetate and lead citrate for 8 min each, then viewed and imaged on a JEM 1220 electron microscope (JEOL, Tokyo, Japan) at 100 kV. To compare collagen fibril density (CFD) between fresh and cryopreserved groups, we analyzed for each group 3 randomly selected transversal section images of collagen fibrils at a magnification of 50,000×. Every image was analyzed with Image J to obtain the number of collagen fibrils and the corresponding area fraction (%) of the collagen fibrils to represent the CFD value [20].

### Apoptosis detection

TUNEL assay was performed on fresh (n=3) and cryopreserved (n=3) lenticules. The lenticules were immersed in OCT (Leica Microsystems, Nussloch, Germany) and frozen at −80 °C until sectioning. Seven-micron thick sections were cut using a MicromHM550 cryostat (Microm, Walldorf, Germany). The TUNEL assay was performed according to manufacturer’s instructions (In Situ Cell Death Detection Kit; Roche Applied Science, Indianapolis, IN). Slides were then mounted with UltraCruz mounting medium containing 4',6-diamidino-2-phenylindole (DAPI; Santa Cruz Biotechnology, Santa Cruz, CA) for nuclear staining [21]. Sections were observed and imaged with Zeiss Axioplan 2 fluorescence microscope (Carl Zeiss, Oberkochen, Germany). The positive TUNEL and DAPI stained cells were manually counted on the peripheral and central area of the lenticules in seven randomly selected fields of each sample using a magnification of 200×.

### In vitro cell viability

Cornea stroma lenticules of fresh (n=3) and cryopreserved (n=3) groups were washed twice in a phosphate-buffered saline (PBS) buffered antibiotic/antimycotic solution and enzymatically digested in serum-supplemented culture medium containing collagenase A (2 mg/ml; Roche, Mannhein, Germany) for at least 2 h. Stromal keratocytes were rinsed twice and seeded onto cell culture dishes coated with Cell Attachment Reagent (FNC Coating Mix, BRFF AF-10; US Biologic, Swampscott, MA). The cells were cultured in Ham’s F12 and M199 (1:1 ratio) medium supplemented with 5% fetal bovine serum (FBS; Invitrogen, Carlsbad, CA), 1× ITS (insulin, transferrin, selenium), 1× antibiotic/antimycotic (all from Invitrogen), 20 μg/ml ascorbic acid (Sigma), and 10 ng/ml bFGF (R&D Systems, Minneapolis, MN). Culture medium was refreshed every two days. All cultures and incubations were performed at 37 °C in a humidified incubator (Binder, Bohemia, NY) at 5% CO_2_.

### Gene expression analysis

RT–PCR was performed for specific keratocyte markers on cells isolated from fresh and cryopreserved lenticules. Briefly, keratocytes were isolated immediately after washing twice (10 min each) with a PBS buffered antibiotic/antimycotic solution, supplemented with 5% FBS and digested with collagenase A (2 mg/ml; Roche) overnight. Cell pellets were rinsed once, snap frozen and stored at −80 °C before use. Total RNA was extracted using a RNeasy kit (Qiagen, Hilden, Germany) and the sample was treated with a DNase and purified using Turbo DNAfree (Ambion, Austin, TX) according to the manufacturers’ instructions. The RNA sample was reverse-transcribed into cDNA using superscript first-strand synthesis system (Invitrogen).

PCR was performed for the expression of human aldehyde dehydrogenase 3A1 (*ALDH3A1*) and Keratocan (*KERA*). For the *ALDH3A1* gene the following primer was used: F=5′-ACT CAG CAG GAC GAG CTC TAC-3′, R=5′-GGG TCA CAG AGG ATG TAG TC-3′ (GenBank NM_001135168.1; Size: 495 bp) [22]. For the *KERA* gene the following primer was used: F=5′-ATC TGC AGC ACC TTC ACC TT-3′, R=5′-CAT TGG AAT TGG TGG TTT GA-3′ (GenBank NM_007035; Size: 167 bp) [23]. PCR was performed alongside a ubiquitously expressed housekeeping gene, Glyceraldehyde 3-phosphate dehydrogenase (*GAPDH*) using the following primer: F=5′-GCC AAG GTC ATC CAT GAC AAC-3′, R=5′-GTC CAC CAC CCT GTT GCT GTA-3′ (GenBank NM_002046.3; Size: 498 bp) [24]. PCR reactions were performed on a thermal cycler (C1000; Bio-Rad Laboratories, Hercules, CA) and followed denaturation at 94 °C for 30 s, primer annealing at 50–60 °C for 30 s, and template elongation at 72 °C for 1 min for 34 cycles, with a final template extension at 72 °C for 5 min. The annealing temperatures used were 58 °C, 52 °C, and 55 °C for *ALDH3A1*, *KERA*, and *GAPDH* primers, respectively. The PCR products were validated via gel electrophoresis on a 1% agarose gel stained with ethidium bromide, and visualized using a Kodak Image Station 4000R (Kodak, Rochester, NY) under ultraviolet light.

### Statistical analysis

All categorical data was analyzed by a two-tailed Pearson χ^2^ test. Continuous data was analyzed by a two-tailed unpaired *t*-test. For same specimen stains, a binominal test was used to test whether it was equally proportioned. Statistics were performed in IBM SPSS v.19 (IBM Corporation, Armonk, NY) software program. Statistically significant data was consider when p<0.05.

## Results

### TEM

**Figure 4:**
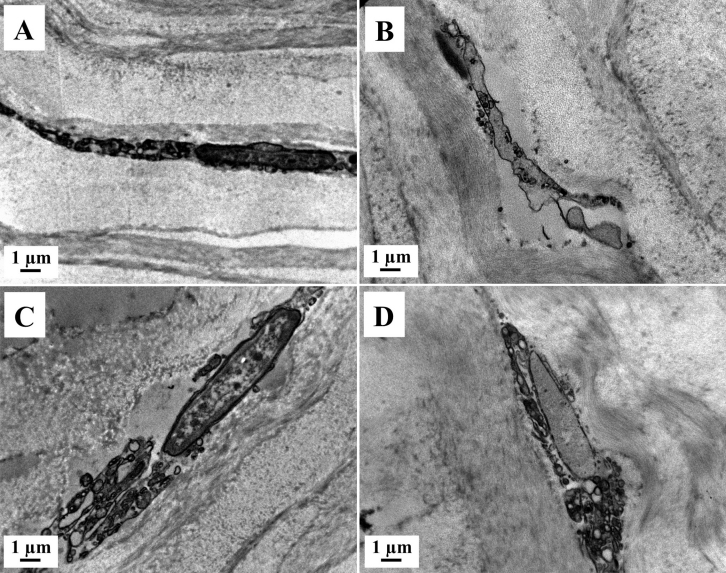
Transmission electron micrographs (TEM) of stromal lenticule showing keratocytes. **A**, **C**: Fresh lenticule. **B**, **D**: Cryopreserved lenticule. **A**, **B**: Apoptotic keratocytes with cromatin condensation and fragmentation, apoptotic bodies, loss of cytoplasm and cell shrinkage. **C**, **D**: Necrotic keratocyte, with incomplete nuclear membrane and vacuoles in the cytoplasm. Magnification, 8900×.

The ultrastructural analysis of fresh (n=3) and cryopreserved (n=3) lenticules with TEM showed apoptotic, necrotic and normal quiescent keratocytes. We observed more necrotic than apoptotic keratocytes, which were mostly located at the periphery of the lenticule (Figure 4).

The normal quiescent keratocytes were defined as those with large nuclei with peripheral heterochromatin and thin cytoplasm with complete cellular and nuclear membrane without visible mitochondria or extensive rough endoplasmic reticulum. Apoptotic keratocytes were defined as having chromatin condensation and fragmentation, shrinkage of nucleus and cell, apoptotic bodies, and/or loss of cytoplasm. Necrotic keratocytes were defined those showing incomplete nuclei and cell membrane, cytoplasmic vacuoles, disperse chromatin with irregular clumpings, and/or swelling and vacuolated nucleus [25].

**Figure 5:**
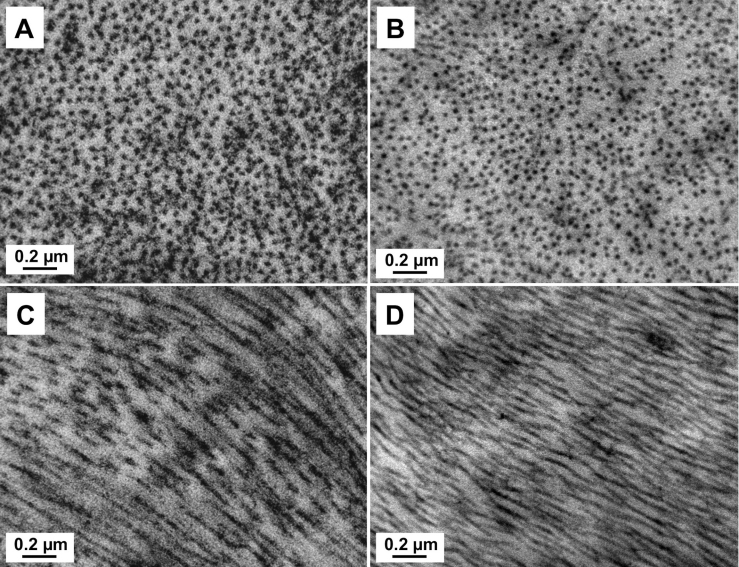
Transmission electron micrographs (TEM) of the stromal lenticule showing collagen fibrils. **A**, **C**: Fresh lenticule. **B**, **D**: Cryopreserved lenticule. **A**, **B**: Transversal section of collagen fibrils. **C**, **D**: Longitudinal section of collagen fibrils. Magnification, 50,000×.

Lenticule collagen fibril architecture in both groups was relatively well preserved after ReLEx and after cryopreservation. There were no fragmented fibrils or areas of collagen disruption; as well as no change in fibrils diameter (Figure 5). The mean (SD) CFD was 15.75±1.56 and 12.05±0.62 for the fresh and cryopreserved group, respectively. The mean (SD) number of collagen fibrils was 728.33±38.19 and 795.33±33.84 in the fresh and cryopreserved groups respectively. There was no significant change in the number of collagen fibrils (p=0.09), but a significant decrease in CFD (p=0.02) was observed after cryopreservation.

### TUNEL assay

**Figure 6:**
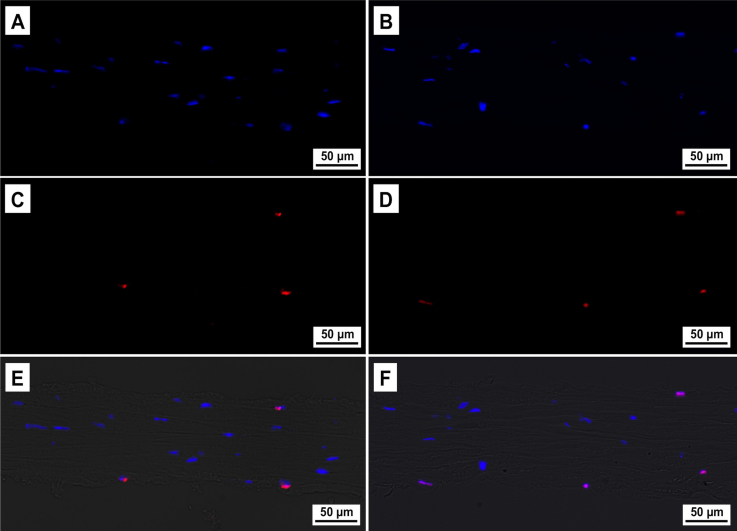
TUNEL-positive (deoxynucleotidyl transferase-mediated nick end labeling assay) cells in fresh and cryopreserved human lenticules. **A**, **C**, **E**: Fresh samples. **B**, **D**, **F**: Cryopreserved samples. **A**, **B**: DAPI-stained (4',6-diamidino-2-phenylindole stain) cells. **C**, **D**: TUNEL-positive cells. **E**, **F**: Composite image of DAPI, TUNEL and Bright-field. Magnification, 200×.

**Figure 7:**
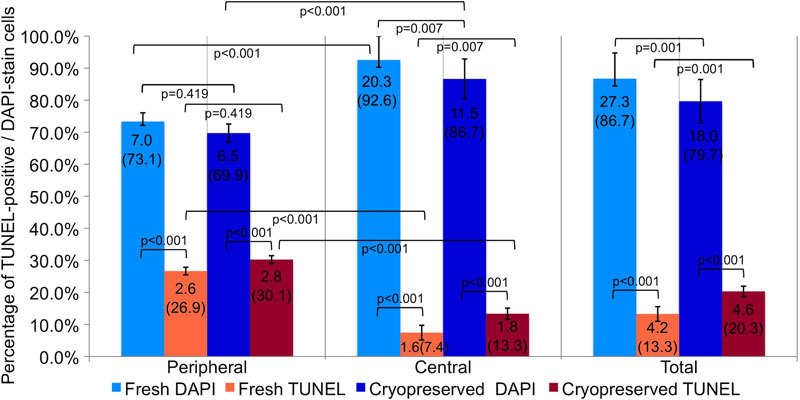
Mean number (%) of TUNEL (deoxynucleotidyl transferase-mediated nick end labeling assay) positive cells and DAPI (4',6-diamidino-2-phenylindole) stain cells in fresh and cryopreserved lenticules extracted from a ReLEx (Refractive Lenticule Extraction) procedure. It was considered a significant difference when p<0.05.

Analysis of apoptotic keratocytes in fresh (n=3) and cryopreserved (n=3) groups was performed using a TUNEL assay, and cell nuclei were counterstained using DAPI (Figure 6). Quantification of TUNEL-positive and DAPI-stained cells is described in Table 1. We observed TUNEL-positive cells in both the groups. However, there was an increase in the number of TUNEL-positive cells and a proportional reduction in the number of DAPI-stained cells after cryopreservation. These findings were significant in the center (p=0.007), but not in the periphery (p=0.419) of the lenticule (Figure 7 and Table 1). Altogether, there were more TUNEL-positive cells located in the periphery than in the center of the lenticules, in fresh (p<0.001) and cryopreserved (p<0.001) groups. The number of DAPI-stained cells was higher in the center than in the periphery, in fresh (p<0.001) and cryopreserved (p<0.001) groups (Figure 7 and Table 1).

**Table 1:** TUNEL and DAPI positive cells in peripheral, central, and total area of fresh and cryopreserved ReLEx lenticules.

**Location area**	**Peripheral**		**Central**		**Total**	
Staining	DAPI	TUNEL	DAPI	TUNEL	DAPI	TUNEL
**Fresh lenticules**						
Total cells	147	54	427	34	574	88
Mean cells	7.0	2.6	20.3	1.6	27.3	4.2
SD	2.73	1.18	7.48	2.28	8.02	2.28
Max - Min	14–2	5–1	35–9	7–0	42–13	9–1
Percentage	73.1	26.9	92.6	7.4	86.7	13.3
**Cryopreserved lenticules**						
Total cells	137	59	242	37	378	96
Mean cells	6.5	2.8	11.5	1.8	18.0	4.6
SD	2.82	1.22	6.22	1.74	6.77	1.62
Max - Min	14–3	5–1	31–1	6–0	42–9	7–1
Percentage	69.9	30.1	86.7	13.3	79.7	20.3

TUNEL: deoxynucleotidyl transferase-mediated nick end labeling assay, DAPI: 4',6-diamidino-2-phenylindole, ReLEx: Refractive Lenticule Extraction procedure, SD: Standard Deviation.

### Cell culture

**Figure 8:**
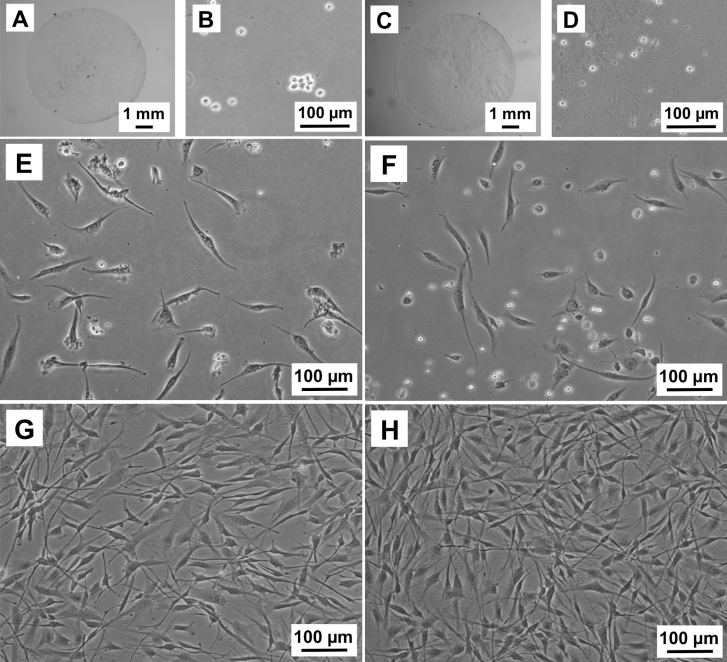
Representative images of cultured keratocytes from ReLEx (Refractive Lenticule Extraction) lenticules. **A**, **B**, **E**, **G**: Fresh samples. **C**, **D**, **F**, **H**: Cryopreserved samples. **A**, **C**: ReLEx lenticule. **B**, **D**: Free floating stromal keratocytes following enzymatic digestion for at least 4 h in collagenase. **E**, **F**: Attached keratocytes beginning to elongate into spindle-like fibroblastic cells by Day 2 in culture. **G**, **H**: Confluent stromal fibroblasts after 7 days in culture.

We were able to culture viable keratocytes from fresh (n=3) and cryopreserved (n=3) lenticules. After stromal digestion with collagenase, isolated keratocytes were cultivated in medium containing serum (FBS). The keratocytes adopted typical elongated morphology of fibroblastic phenotype within two days in culture and rapidly proliferated to reach confluence in one week, in both the groups. We did not notice any difference in cellular morphology or proliferation rates between the groups (Figure 8).

### RT–PCR

**Figure 9:**
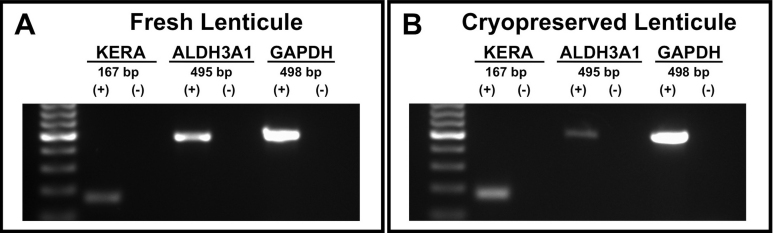
Expression of keratocyte-specific markers in isolated cells from ReLEx (Refractive Lenticule Extraction) lenticules. Fresh (**A**) and cryopreserved (**B**) lenticues. Human keratocan (*KERA*) with 167 bp, aldehyde dehydrogenase 3A1 (*ALDH3A1*) with 495 bp and the housekeeping gene glyceraldehyde 3-phosphate dehydrogenase (*GAPDH*) with 498 bp. (+): Lenticule sample. (-): Negative control.

Gene expression analysis of fresh and cryopreserved lenticules showed expression of keratocyte-specific genes *KERA* and *ALDH3A1* (Figure 9).

## Discussion

In the present study, we evaluated the viability of human cornea lenticules extracted following ReLEx procedure and one month after storage using a developed cryopreservation technique. Our TEM findings showed similar pattern of apoptotic and quiescent keratocytes within a well preserved and well aligned collagen structure in fresh and cryopreserved lenticules. Similarly, TUNEL-positive cells were mainly localized in the periphery of the lenticule in both groups. However, there was a significant increase in the TUNEL positive cells in the center of the lenticule following cryopreservation. We were able to verify the presence of keratocyte-specific gene expression of *KERA* and *ALDH3A1* by RT–PCR in the cells isolated from the lenticules both pre and post cryopreservation. The viability of the cells in fresh and cryopreserved lenticules was also confirmed by cell culture. The differences in donor age, time from death to tissue harvest and time from death to surgery in our sample should not significantly affect our findings. It has been reported previously that there is no correlation between the donor age, death to preservation time or total storage time of the corneas and the percentage of dead keratocytes when stored in Optisol [26]. International donor sharing and air-transportation of corneas stored in Optisol is as safe and effective as using local tissue [27-29].

The cryopreservation of the extracted stromal lenticule was achieved using a cryopreservation procedure that involved gradual cooling of the stromal lenticule (in a cryocontainer) to −80 °C in the presence of a cryoprotectant, DMSO, before being transferred into liquid nitrogen for long-term storage [30]. This approach is known to lessen the damage caused by intracellular ice formation [30]. In this study, viable keratocytes were isolated from the cryopreserved stromal lenticule, and expanded into healthy stromal fibroblasts. Similar cryopreservation techniques have been successfully used in the storage of cornea and other human tissues [31]. However, standard cryopreservation and freeze–thaw techniques (e.g., liquid nitrogen [15,32], freezing at −80 °C [33], nitrogen (N_2_) gas [34], and cryoprobe at −80 °C [19]) have been shown to induce apoptosis and necrosis in keratocytes [31,35]. Differential inter-species variation with respect to collagen fibril damage has also been shown following cryopreservation [15]. In the present study, TEM analysis showed a decrease in CFD after cryopreservation without a significant change in the number of collagen fibrils. This change is due to the swelling of tissues that takes place after cryopreservation. However, overall collagen integrity and structure was maintained in the cryopreserved lenticules. Collagen fibrils remained well preserved and well aligned, similar to the freshly extracted lenticules after ReLEx procedure. These observations confirm that our cryopreservation technique with the lenticules is reliable in maintaining the collagen structure of the lenticule. Regular collagen architecture is one of the key factors to maintain cornea transparency [36]. Therefore, if the lenticule is to be considered for re-implantation, maintenance of regular corneal collagen architecture is vital following cryopreservation.

TUNEL positive cells were detected mainly in the periphery of the fresh lenticules. These results are similar to our previous report on ReLEx in rabbits [12], where there was large amount of TUNEL-positive cells in the periphery of unextracted lenticules [12]. This result implied that the peripheral damage was produced by FS laser and not by the manual removal of the lenticule. However, there was an increase in the amount of TUNEL-positive cells in the center of the lenticules following cryopreservation but no significant increase in the periphery. Our results suggest that the cells located in the center of the lenticule are more susceptible to damage during cryopreservation and thawing process.

Transmission electron microscopy (TEM) showed more necrotic than apoptotic keratocytes in both the fresh and cryopreserved groups. Several reports have shown mixed necrotic [32] or apoptotic [15] keratocytes following liquid nitrogen cryopreservation. One of the limitations of TUNEL assay is that it detects fragmented DNA, which is not entirely specific for apoptotic cells [37-40]. In fact, keratocytes undergoing necrosis after FS laser have been recognized by TUNEL assay [41]. In addition, we noted a significant decrease in the number of DAPI positive, TUNEL negative cells in the center of the lenticule following cryopreservation. This maybe due to the following mechanisms: 1) TUNEL assay is less sensitive to the single-strand DNA breaks in late stage necrosis than the double stranded DNA fragmentation in apoptosis [40] and 2) DAPI visualize cells by specifically staining the nuclei [21] and in late necrosis some nuclei may not stain. Hence we hypotheses that the reduction in number of DAPI positive (TUNEL negative cells) and the main mode of keratocyte death in fresh and cryopreserved lenticules following ReLEx is most likely to cell necrosis as opposed to apoptosis.

Isolated cells from fresh and cryopreserved lenticules showed a positive expression of KERA and ALDH3A1. KERA, a small leucine-rich proteoglycan, is a specific marker for keratocytes and highly expressed in the corneal stroma [42]. It is associated with the production of extracellular matrix and aids in corneal transparency [36]. The corneal crystallin, ALDH3A1 is a water-soluble protein highly expressed in quiescent keratocytes, and is associated with maintenance of corneal transparency [43-46]. Thus, the expression of these specific keratocyte markers in the fresh and cryopreserved lenticules showed that they contain viable and quiescent keratocytes.

Keratocytes isolated and cultured from both fresh and cryopreserved lenticules showed similar growth dynamics, taking on a fibroblastic phenotype and proliferating rapidly after growth in serum containing culture medium [36,43,44,47]. More importantly, our results suggests that although dead keratocytes were seen using TEM and TUNEL assay, there were enough viable keratocytes within the cryopreserved lenticules that could be isolated and propagated following 1-month storage. Similar findings have also been previously described following cryopreservation of whole human corneas [33] and freeze–thaw injury in pig corneas [15].

There are several clinical implications of our study. The viability of long-term lenticule storage by cryopreservation after ReLEx surgery may offer an added dimension to corneal refractive laser surgery, that of potential reversibility. Lenticules may be stored for individual ReLEx patients for a prolonged period of time, and could conceivably be re-implanted at a later date in the event of keratectasia, or even as a means of treating presbyopia several years later, by restoring myopia in the non-dominant eye to create a state of monovision, or as an autologous presbyopic lenticular inlay. In addition, with appropriate informed consent, as well as serology clearance, these lenticules could be used as allograft refractive lenticules in other patients who develop post-LASIK keratectasia, in other forms of keratectasia, such as keratoconus, and also may be used as allograft presbyopic inlays, as it is well known that minimal risks of immune-mediated allograft rejection occur in anterior lamellar keratoplasty [48].

In summary, the stromal lenticule extracted following ReLEx maintain keratocyte viability and overall collagen structural integrity in pre- and post- cryopreserved tissue samples. Keratocytes have been shown to be an important contributor for the maintenance of corneal transparency [36,43,44,46,47] and this may be important if the lenticule is to be re-implanted in future e.g., in the treatment of corneal ectasia following myopic correction. However, the maintenance of the collagen architecture is probably the most important finding since corneal stromal buttons decellularized of keratocytes have been shown to be viable following host keratocyte migration [34]. Future work evaluating the viability of these lenticules following reimplantation is currently being investigated using an in vivo animal model of ReLEx.

## References

[1] Vaddavalli PK, Yoo SH (2011). Femtosecond laser in-situ keratomileusis flap configurations.. Curr Opin Ophthalmol.

[2] Tran DB, Binder PS, Brame CL (2008). LASIK flap revision using the IntraLase femtosecond laser.. Int Ophthalmol Clin.

[3] Kunert KS, Blum M, Duncker GIW, Sietmann R, Heichel J (2011). Surface quality of human corneal lenticules after femtosecond laser surgery for myopia comparing different laser parameters.. Graefes Arch Clin Exp Ophthalmol.

[4] Espandar L, Meyer J (2010). Intraoperative and Postoperative Complications of Laser in situ Keratomileusis Flap Creation Using IntraLase Femtosecond Laser and Mechanical Microkeratomes.. Middle East Afr J Ophthalmol.

[5] Kim P, Sutton GL, Rootman DS (2011). Applications of the femtosecond laser in corneal refractive surgery.. Curr Opin Ophthalmol.

[6] Stonecipher K, Ignacio TS, Stonecipher M (2006). Advances in refractive surgery: microkeratome and femtosecond laser flap creation in relation to safety, efficacy, predictability, and biomechanical stability.. Curr Opin Ophthalmol.

[7] Ahn H, Kim JK, Kim CK, Han GH, Seo KY, Kim EK, Kim TI (2011). Comparison of laser in situ keratomileusis flaps created by 3 femtosecond lasers and a microkeratome.. J Cataract Refract Surg.

[8] Sekundo W, Kunert K, Russmann C, Gille A, Bissmann W, Stobrawa G, Sticker M, Bischoff M, Blum M (2008). First efficacy and safety study of femtosecond lenticule extraction for the correction of myopia: six-month results.. J Cataract Refract Surg.

[9] Blum M, Kunert KS, Engelbrecht C, Dawczynski J, Sekundo W (2010). Femtosecond lenticule extraction (FLEx) – Results after 12 months in myopic astigmatism.. Klin Monatsbl Augenheilkd.

[10] Blum M, Kunert K, Schröder M, Sekundo W (2010). Femtosecond lenticule extraction for the correction of myopia: preliminary 6-month results.. Graefes Arch Clin Exp Ophthalmol.

[11] Sekundo W, Kunert KS, Blum M (2011). Small incision corneal refractive surgery using the small incision lenticule extraction (SMILE) procedure for the correction of myopia and myopic astigmatism: results of a 6 month prospective study.. Br J Ophthalmol.

[12] Riau AK, Angunawela RI, Chaurasia SS, Lee WS, Tan DT, Mehta JS (2011). Early corneal wound healing and inflammatory responses following Refractive Lenticule Extraction (ReLEx).. Invest Ophthalmol Vis Sci.

[13] Capella JA, Kaufmann HE, Robbins JE (1965). Preservation of viable corneal tissue.. Cryobiology.

[14] Eastcott HH, Cross AG, Leigh AG, North DP (1954). Preservation of corneal grafts by freezing.. Lancet.

[15] Oh JY, Kim MK, Lee HJ, Ko JH, Wee WR, Lee JH (2009). Comparative observation of freeze–thaw-induced damage in pig, rabbit, and human corneal stroma.. Vet Ophthalmol.

[16] Neronov A, Mazgalova J, Cholakova M, Dimitrova M, Deligiozova I, Kovatcheva S, Nikolova E (2005). Integrity of endothelium in cryopreserved human cornea.. Cryo Lett.

[17] Halberstadt M, Böhnke M, Athmann S, Hagenah M (2003). Cryopreservation of human donor corneas with dextran.. Invest Ophthalmol Vis Sci.

[18] Halberstadt M, Athmann S, Hagenah M (2001). Corneal cryopreservation with dextran.. Cryobiology.

[19] Oh JY, Lee HJ, Khwarg SI, Wee WR (2010). Corneal cell viability and structure after transcorneal freezing-thawing in the human cornea.. Clin Ophthalmol.

[20] Sanders JE, Goldstein BS (2001). Collagen fibril diameters increase and fibril densities decrease in skin subjected to repetitive compressive and shear stresses.. J Biomech.

[21] Tarnowski BI, Spinale FG, Nicholson JH (1991). DAPI as a useful stain for nuclear quantitation.. Biotech Histochem.

[22] Du Y, Roh DS, Funderburgh ML, Mann MM, Marra KG, Rubin JP, Li X, Funderburgh JL (2010). Adipose-derived stem cells differentiate to keratocytes in vitro.. Mol Vis.

[23] Pei Y, Reins R, McDermott A (2006). ALDH3A1 Expression by the human keratocyte and its repair phenotypes.. Exp Eye Res.

[24] Lu X, Chen D, Liu Z, Li C, Liu Y, Zhou J, Wan P, Mou YG, Wang Z (2010). Enhanced survival in vitro of human corneal endothelial cells using mouse embryonic stem cell conditioned medium.. Mol Vis.

[25] Komuro A, Hodge DO, Gores GJ, Bourne WM (1999). Cell death during corneal storage at 4 degrees C.. Invest Ophthalmol Vis Sci.

[26] Komuro A, Hodge DO, Gores GJ, Bourne WM (1999). Cell death during corneal storage at 4 C.. Invest Ophthalmol Vis Sci.

[27] Halberstadt M, Athmann S, Winter R, Hagenah M (2000). Impact of transportation on short-term preserved corneas preserved in Optisol-GS, Likorol, Likorol-DX, and MK-medium.. Cornea.

[28] Varssano D, Russ V, Linhart Y, Lazar M (2005). Air transportation of corneal tissue: experience with local compared to transatlantic donor corneas.. Cornea.

[29] Shimazaki J, Shinozaki N, Shimmura S, Holland EJ, Tsubota K (2004). Efficacy and Safety of International Donor Sharing: A Single-Center, Case-Controlled Study on Corneal Transplantation.. Transplantation.

[30] Hunt CJ (2011). Cryopreservation of Human Stem Cells for Clinical Application: A Review.. Transfus Med Hemother.

[31] Baust JG, Gao D, Baust JM (2009). Cryopreservation: An emerging paradigm change.. Organogenesis.

[32] Villalba R, Peña J, Luque E, Villalba JM, Gómez-Villagrán JL (2004). Keratocyte injury in human corneas cryopreserved under standard conditions.. Cell Tissue Bank.

[33] Borderie VM, Lopez M, Lombet A, Carvajal-Gonzalez S, Cywiner C, Laroche L (1998). Cryopreservation and culture of human corneal keratocytes.. Invest Ophthalmol Vis Sci.

[34] Amano S, Shimomura N, Yokoo S, Araki-Sasaki K, Yamagami S (2008). Decellularizing corneal stroma using N2 gas.. Mol Vis.

[35] Baust JM (2002). Molecular mechanisms of cellular demise associated with cryopreservation failure.. Cell Preserv Technol.

[36] Hassell JR, Birk D (2010). The molecular basis of corneal transparency.. Exp Eye Res.

[37] Loo DT (2011). In situ detection of apoptosis by the TUNEL assay: an overview of techniques.. Methods Mol Biol.

[38] Kumari S, Rastogi RP, Singh KL, Singh SP, Sinha RP (2008). DNA damage: detection strategies.. Excli Journal.

[39] Grasl-Kraupp B, Ruttkay-Nedecky B, Koudelka H, Bukowska K, Bursch W, Schulte-Hermann R (1995). In situ detection of fragmented DNA (TUNEL assay) fails to discriminate among apoptosis, necrosis, and autolytic cell death: a cautionary note.. Hepatology.

[40] Hewitson TD, Bisucci T, Darby IA (2006). Histochemical localization of apoptosis with in situ labeling of fragmented DNA.. Methods Mol Biol.

[41] Netto MV, Mohan RR, Medeiros FW, Dupps WJ, Sinha S, Krueger RR, Stapleton WM, Rayborn M, Suto C, Wilson SE (2007). Femtosecond laser and microkeratome corneal flaps: comparison of stromal wound healing and inflammation.. J Refract Surg.

[42] Carlson EC, Liu C-Y, Chikama T-i, Hayashi Y, Kao CW-C, Birk DE, Funderburgh JL, Jester JV, Kao WW (2005). Keratocan, a cornea-specific keratan sulfate proteoglycan, is regulated by lumican.. J Biol Chem.

[43] Pei Y, Reins RY, McDermott AM (2006). Aldehyde dehydrogenase (ALDH) 3A1 expression by the human keratocyte and its repair phenotypes.. Exp Eye Res.

[44] West-Mays JA, Dwivedi DJ (2006). The keratocyte: Corneal stromal cell with variable repair phenotypes.. Int J Biochem Cell Biol.

[45] Jester JV, Moller-Pedersen T, Huang J, Sax CM, Kays WT, Cavangh HD, Petroll WM, Piatigorsky J (1999). The cellular basis of corneal transparency: evidence for ‘corneal crystallins’.. J Cell Sci.

[46] Jester JV (2008). Corneal Crystallins and the Development of Cellular Transparency.. Semin Cell Dev Biol.

[47] Fini ME, Stramer BM (2005). How the cornea heals: cornea-specific repair mechanisms affecting surgical outcomes.. Cornea.

[48] Luengo-Gimeno F, Tan DT, Mehta JS (2011). Evolution of deep anterior lamellar keratoplasty (DALK).. Ocul Surf.

